# A Case of Late-Onset Local Anesthetic Toxicity Observed as Seizure Activity

**DOI:** 10.7759/cureus.25649

**Published:** 2022-06-04

**Authors:** Ahmet Salih Tüzen, Derya Arslan Yurtlu, Ahmet Said Çetinkaya, Murat Aksun, Nagihan Karahan

**Affiliations:** 1 Department of Anesthesiology and Reanimation, Izmir Katip Celebi University Atatürk Training and Research Hospital, Izmir, TUR

**Keywords:** seizure risk, systemic toxicity, local anesthetic toxicity, infraclavicular brachial plexus block, brachial plexus block

## Abstract

Most of the local anesthetic toxicity cases develop within the first five minutes of peripheral block administration. Late local anesthetic toxicity has been rarely reported in the literature. However, it is an important life-threatening problem that can lead to seizures, hemodynamic collapse, and cardiac arrest if it is ignored and not considered. Here we present the case of an 18-year-old male patient who had ultrasonography-guided infraclavicular brachial plexus block administration with a 30 mL local anesthetic. The patient had convulsions 210 minutes after the block administration and was treated with intravenous diazepam. Intraoperative and postoperative courses were uneventful. He had no neurologic signs or symptoms afterward. All laboratory tests and radiologic investigation tests were normal. This report demonstrates that late local anesthetic toxicity is still possible after several hours of the uneventful peripheral neural blockade, although it is rarely reported.

## Introduction

Although local anesthetic toxicity is observed rarely, it is notable as one of the complications of peripheral nerve blockade with the highest mortality. Toxicity is generally caused by mistaken local anesthetic administration into the systemic circulation, rapid systemic absorption, or administration of amounts above the safe limits. In 75% of reported cases, toxicity is observed in the first five minutes of anesthetic administration. This early onset is due to inadvertent intravascular injection generally progressing with isolated central nervous system involvement; however, the cardiovascular system is affected in serious cases [1]. In this report, we aim to present a case involving a brachial plexus block administered with the infraclavicular approach that led to generalized tonic-clonic seizure activity at postoperative 3.5 hours leading to the consideration of late-onset local anesthetic toxicity.

## Case presentation

An 18-year-old male patient, with 75 kg weight and 175 cm height, underwent an internal fixation operation for a distal radius fracture with planned administration of a brachial plexus block with the infraclavicular technique. The patient had no prior history of epilepsy and smoking, alcohol or drug use, no comorbid diseases or anesthesia history, and had normal laboratory tests; thus, he was assessed as in American Society of Anesthesiologists (ASA) physical status I. Detailed information was given explaining the procedure and written consent was obtained from the patient. Premedication was not administered to maintain cooperation during the procedure.

The patient was monitored in the operating room; his vital signs were within the normal limits. After skin antisepsis, ultrasonography was performed (SonoSite M-Turbo; Fujifilm SonoSite Inc., Bothell, WA); a 10-18 MHz linear probe was placed in the cavity between the clavicle and coracoid promontory, and arterial pulsation was observed. The puncture point was determined 0.5 cm below the clavicula.

A local anesthetic mixture was prepared with 15 mL of 0.5% bupivacaine (5 mg/mL; Marcaine; AstraZeneca, Istanbul) and 15 mL of 2% prilocaine (20 mg/mL; Citanest; AstraZeneca). A 21-gauge 100-mm Stimuplex A needle (Braun, Melsungen, Germany) was directed to the lower section of the artery with the in-plane technique following the whole path of the needle from the skin. Aspiration was performed before injection, and after observing distribution in the region targeted for local anesthetic distribution with a test dose, the injection was begun. With repeated aspiration after every 5 mL, the injection continued. The injection was completed with a total of 30 mL of the local anesthetic. Local anesthetic distribution was observed in all cords. During injection, full communication was maintained with the patient; his vital signs were stable and no abnormality was observed. After injection was completed, motor block began at the 15th minute, and sufficient anesthesia for surgery was then provided. Then 2 mg intravenous (IV) midazolam (Dormicum; Deva Holding, Istanbul) was administered and he was taken to the operation theatre.

During the operation lasting nearly 1.5 hours, the patient had stable vital signs and did not require sedation or analgesia. The patient’s hemodynamic status was stable in the post-anesthesia care unit (PACU). He was oriented, cooperative, and pain-free when transferred to the orthopedic ward in the third hour. At the 30th minute of the patient’s arrival to the ward, he suddenly lost consciousness accompanied by shivering, convulsions, and generalized tonic-clonic seizure activity lasting three minutes. Intravenous diazepam was administered by the ward staff immediately. When the patient regained consciousness, the neurologic examination did not identify any abnormal findings. In the postictal period, blood pressure was 129/55 mmHg, heart rate 114/min, respiration 15/min and peripheral oxygen saturation at 97%, with the physical examination revealing butterfly-style erythematous appearance on the face. Serum bupivacaine and prilocaine levels were not studied in our center. Therefore, we could not determine serum blood levels. In addition, the seizure activity had ended five minutes before the patient was reached. Therefore, we did not give lipid infusion as no abnormal findings were detected with careful hemodynamic monitoring. In addition, continuous positive airway pressure ventilation was not performed because acidosis and hypercapnia were not detected in arterial blood gas analysis. On the recommendation of a neurologist, brain tomography was taken on the same day and moderate hyperdensity was observed in the left transverse sinus of the patient; however, diffusion magnetic resonance imaging (MRI) did not identify significant diffusion limitation. Brain venography and electroencephalography (EEG) results did not identify significant pathology and the patient was discharged after two days of problem-free observation on the ward. The patient didn’t have any further seizure episodes within the one-year follow-up.

## Discussion

Local anesthetic toxicity results from high plasma medication concentrations. The most important risk factors for local anesthesia toxicity are accidental intravascular injection, drug dose above the safe limits, and rapid systemic absorption [2]. The close proximity of peripheral nerves to vascular structures increases the risk of accidental intravascular injection and especially causes rapidly progressive severe toxicity findings.

Sudden increases in the local anesthetic concentration in the systemic circulation cause the earlier occurrence of toxic effects in well-perfused organs like the brain and heart [3]. Toxicity symptoms generally display as tinnitus, perioral numbness, dizziness, metallic taste on the tongue, discomfort from visual-verbal stimuli, confusion, agitation, seizure, and reduced consciousness in the central nervous system. Effects on the cardiovascular system include dysrhythmia, conduction disorder, tachycardia, and hypertension, with myocardial depression hypotension, bradycardia, and cardiovascular collapse with cardiac arrest in advanced periods [4].

While toxicity symptoms occur in the first 50 seconds in 50% of cases, it was revealed that 75% of cases had clinical findings of toxicity symptoms within the first five minutes [1]. The incidence of early-onset local anesthetic toxicity varies from 0.04% to 0.18%, while late-onset toxicity findings are encountered more rarely in the literature [5-9].

Factors affecting absorption of the local anesthetic are considered to play a greater role in late-onset local anesthesia administration. Among the factors affecting the absorption rate are choice of medication, medication dose, administration speed, presence of adjuvant vasoactive medications, age, patient comorbidities and vascularity in the perineural area, blood flow rate, local tissue binding, and lipophilicity [3]. Especially, aminoamide-group local anesthetics may show individual variations in clinical effects linked to transport by alpha-1-acid glycoprotein and first transition elimination of cytochrome p450 enzymes in the liver [10]. The low hepatic enzyme levels and low alpha-1-acid glycoprotein levels in infants and pregnant women increase the free drug fraction and may cause the development of toxicity.

Agitation and convulsions observed in our patient occurred 3.5 hours after local anesthetic administration for the infraclavicular block. The total doses of 75 mg bupivacaine and 300 mg prilocaine were below the recommended toxic dose limits. The additive effect of the use of combined local anesthetics may be a risk factor in terms of toxicity. In toxicity-developed cases, the rate of bupivacaine use is higher than other agents [1,10]. Toxicity may develop against prilocaine; however, the chances are reduced by more rapid metabolism and a larger safety interval [2]. In our case, the combination of bupivacaine and prilocaine was used, and the additive effect cannot explain the toxicity findings developing in the long term. There were no symptoms of systemic disease, medication, or drug use that could explain the late toxicity symptoms observed in this patient.

A range of precautions is available in order to reduce the risk of toxicity in central and peripheral block administration. These include individualized dose, slow and intermittent medication administration, aspiration before injection, observation of the local anesthetic filling the injection area, administration of a low-concentration epinephrine test dose and observations of variations in heart rate and blood pressure, premedication with medications like benzodiazepines preventing seizure activity, continuous communication with the patient before deep sedation induction and awareness and intervention for early clinical findings of toxicity [11].

The use of ultrasound compared to paresthesia or nerve stimulators for peripheral nerve blocks reduced the incidence of toxicity by 60%. The advantages of ultrasound use are direct imaging of the nerves, performing the injection by observing all anatomic structures, observation of needle progression and tip to clarify the injection area, reducing the dose by observing the distribution of the local anesthetic administered, reducing the number of needle orientations and increasing patient comfort [12,13]. In this case, ultrasound was used to observe the vascular structures, and aspiration was used to check that there was no blood flow before local anesthetic injection. However, even with blocks administered with ultrasound observation, systemic toxicity cases are reported after IV administration of local anesthetics. In spite of no venous puncture, cases developing convulsions are reported [14].

In situations where the cardiovascular system is affected, it is necessary to begin treatment with IV lipid solutions immediately to increase the success of resuscitation [9]. Most practitioners may consider administering lipid infusion in order to prevent cardiovascular system involvement in a seizure patient. However, seizure activity ended in a short period in our case and lipid treatment was not required as hemodynamic parameters were stable. The first approaches to prevent seizure activity should be benzodiazepines, barbiturates, and if necessary, propofol administration. In our case, IV diazepam was administered for seizure activity and the seizure successfully ended. However, lower thresholds for intubation and positive pressure ventilation may be required to prevent hypoxia and acidosis caused by prolonged seizure activity from exacerbating local anesthetic toxicity [15].

## Conclusions

The most feared complication of a peripheral nerve block is local anesthetic toxicity that may develop in the early period; however, toxicity cases beginning in the late period are also encountered. As a result, it should not be forgotten that toxicity may develop in the late period including the postoperative period, and patients need to be adequately monitored for local anesthetic toxicity in the postoperative period. Clinical findings with faint progression should be definitely carefully assessed and correlated with the serum assay of local anesthetic levels. Communication should be maintained with the patient and necessary treatment approaches should be applied when suspicions are raised during monitoring.
